# An Efficient Relayed Broadcasting Based on the Duplication Estimation Model for IoT Applications

**DOI:** 10.3390/s19092038

**Published:** 2019-04-30

**Authors:** Youngboo Kim, Eun-Chan Park

**Affiliations:** Department of Information and Communication Engineering, Dongguk University-Seoul, Seoul 04620, Korea; 0bookim@dongguk.edu

**Keywords:** broadcast storm, flooding, relay, reliability, IoT applications

## Abstract

In this paper, we consider relay-based broadcasting in wireless ad hoc networks, which can enable various emerging services in the Internet of Things (IoT). In this kind of traffic dissemination scheme, also known as flooding, all the nodes not only receive frames but also rebroadcast them. However, without an appropriate relay suppression, a broadcast storm problem arises, i.e., the transmission may fail due to severe collisions and/or interference, many duplicate frames are unnecessarily transmitted, and the traffic dissemination time increases. To mitigate the broadcast storm problem, we propose a reasonable criterion to restrict the rebroadcasting named the duplication ratio. Based on this, we propose an efficient mechanism consisting of duplication suppression and re-queuing schemes. The former discards duplicate frames proactively in a probabilistic manner to decrease the redundancy whereas the latter provides a secondary transmission opportunity reactively to compensate for the delivery failure. Moreover, to apply the duplication ratio practically, we propose a simple method to approximate it based on the number of adjacent nodes. The simulation study confirms that the proposed mechanism tightly ensured the reliability and decreased the traffic dissemination time by up to 6-fold compared to conventional mechanisms.

## 1. Introduction

**Figure 1:**
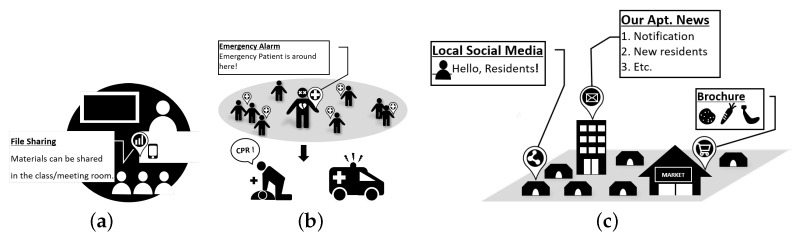
Examples of IoT services based on relayed broadcasting: (**a**) file sharing, (**b**) an emergency alarm, and (**c**) local social media or marketing.

The recent advances in Internet of Things (IoT) technologies have introduced many emerging applications [1,2,3,4]. We consider that a relay-based broadcasting is one of the key enabling technologies for IoT services, which can be used to propagate traffic to many unspecified nodes located beyond the transmission range of a source node. In this type of traffic spreading called flooding or relayed broadcasting  (In this study, we use two terms: “flooding” and “relayed broadcasting” interchangeably), all of the nodes work as receivers and at the same time, rebroadcast the received frames to adjacent nodes. In particular, we consider several typical IoT services (data service, public safety [5], and social network [6]) that can be realized by the relayed broadcasting as shown in Figure 1. For example, files can be distributed or shared among a large number of users in a conference hall (Figure 1a) or an emergency alarm can be propagated to many users to provide immediate first aid in a hospital or disaster relief environment (Figure 1b). As shown in Figure 1c, the flooding can also be used to provide social messages or location-based marketing information to users in a community or shopping mall. In addition, the flooding can be effectively used to disseminate traffic in wireless sensor networks (WSN) and vehicular ad hoc networks or in device-to-device communication [7,8]. A common feature of these services is that the data should be delivered in a timely manner to as many users as possible, even if the network is temporarily set up without the aid of an appropriate infrastructure. Meanwhile, most IoT devices are equipped with the wireless local area network (WLAN) interface [9] and their number is growing continuously. They operate in unlicensed bands which are free of charge. As a result, it can be expected that the flooding in WLANs can be an attractive and cost-effective way to enable various IoT services.

Despite the various advantages of flooding, it is faced with a fundamental problem in that a large number of duplicate frames are unnecessarily transmitted, which can lead to severe collisions and/or interference; this is referred to as a *broadcast storm problem* [10]. Accordingly, the reliability of the frame transmission degrades and the dissemination time of the traffic increases. The primary cause of this problem is the absence of a centralized control mechanism, which incurs a significant signaling overhead and is difficult to implement in WLANs. Furthermore, as the density of the nodes increases, this problem becomes worse if the transmission is not properly suppressed. To solve this problem, it is essential to establish a reasonable criterion for suppressing the transmission of duplicate frames. Various criteria have been proposed in the literature, like a threshold value for the number of duplicate frames or the probability depending on the number of adjacent nodes. However, they have not successfully dealt with the trade-off between transmission reliability and traffic dissemination time because they could not tightly ensure the reliability without increasing the delivery time or they intended to decrease the traffic dissemination time at the cost of reliability. Furthermore, the values of these criteria were set in a heuristic way without systematic and theoretical grounds and it is difficult to estimate their optimal values in various dynamic network environments.

In this paper, we introduce an effective criterion for flooding suppression called the *duplication ratio* to provide the reliable and fast propagation of traffic. The duplication ratio is related to the probability that adjacent nodes have already received a specific frame, and it is modeled as a function of the number of duplicate receptions. It is used to determine the probability that each node attempts or abandons the relaying of a frame, thus the flooding of frames is suppressed in a distributed and probabilistic manner depending on the duplication ratio. It is possible that the transmission of a certain frame is excessively suppressed or it fails due to a collision with or interference from concurrent transmissions. To cope with this problem, we propose a *re-queuing* scheme that selects a frame to compensate for excessive suppression or to recover a transmission failure and provides a secondary transmission opportunity for the selected frame. Therefore, the re-queuing scheme effectively improves the reliability of flooding. Furthermore, we propose a simple method to approximate the duplication ratio, which is difficult to estimate accurately without an appropriate acknowledgment mechanism for broadcasting. Simulation results showed that the proposed mechanism successfully delivered the data frame of a source node approximately 99% to all of the nodes and it significantly decreased the traffic dissemination time by up to 6-fold compared to the conventional flooding mechanism focusing on the transmission reliability. The notion of the duplication ratio was first proposed in our previous study [11]. In the present work, we significantly extend our previous study by investigating the characteristics of the duplication ratio in detail and using it in the re-queuing scheme, which was not considered in our previous work. Furthermore, we evaluate and compare the performance of the proposed mechanism in depth by considering diverse simulation configurations and performance indices. The contributions of our work are summarized as follows:

- We present the duplication ratio as a systematic and unified criterion for relay suppression and compensation. We also propose a simple and practical method to approximate it.
- As well as decreasing the redundancy, the proposed mechanism improves the reliability of flooding by combining duplication suppression and re-queuing schemes.
- The proposed mechanism is totally decoupled from the channel access mechanism of WLAN and works in a distributed manner without incurring a signaling overhead or relying on a feedback mechanism. Moreover, it does not require adjusting control parameters depending on network conditions.

The rest of the paper is organized as follows. Section 2 contains a statement of the problem of the flooding mechanism and a discussion of related work in the literature. We introduce the duplication ratio and propose the relay suppression and re-queuing schemes in Section 3. In Section 4, we evaluate and compare the performance of the proposed mechanism via simulations. Finally, we conclude the paper in Section 5.

## 2. Statement of the Problem

### 2.1. Motivation and Challenges

**Figure 2:**
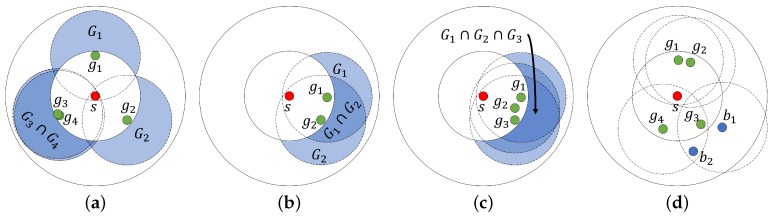
The broadcast storm problem under various network statuses: (**a**) non-overlap and full-overlap, (**b**) partial overlap (two nodes), (**c**) partial overlap (three nodes), and (**d**) collisions and interference.

First, we make the following assumptions about flooding considered in this study; (i) there does not exist any central node that collects global information about the network topology and controls the relay operation of nodes, (ii) each node is unaware of the locations of its adjacent nodes and it does not maintain a list of adjacent nodes, (iii) any control frames including an acknowledgment (ACK) frame are not advertised to or exchanged between adjacent nodes. Figure 2 illustrates several problems that can arise in flooding: redundancy, unreliability, collisions, and interference. The node $g_{i}$ is defined as a node that can receive frames from a source node *s* and is denoted as a *1-hop node*. The area $G_{i}$ is defined as the transmission coverage of node $g_{i}$ where the frames can be propagated via the relaying of $g_{i}$ to the adjacent nodes (denoted as *2-hop nodes*) that cannot directly receive frames from the source node. First, Figure 2a shows two cases: *non-overlap* and *full-overlap*. In the former case, $G_{1}$ and $G_{2}$ do not overlap at all, i.e., any 2-hop node located in $G_{1}$ or $G_{2}$ does not receive a duplicate frame even though both $g_{1}$ and $g_{2}$ broadcast the same frame. Subsequently, it is necessary not to restrict the transmissions of $g_{1}$ and $g_{2}$. On the other hand, in the full-overlap case where $G_{3}$ and $G_{4}$ overlap entirely and regardless of which node (i.e., $g_{3}$ or $g_{4}$) first transmits, any subsequent transmission will result in the unnecessarily duplicate reception at all of the 2-hop nodes in $G_{3}=G_{4}$. The problem in this case can be easily avoided by restricting the redundant transmission of the node detecting the transmission of the same frame.

However, the problem is not easy to deal with in the case of *partial-overlap* where $G_{1}$ and $G_{2}$ partially overlap (Figure 2b) because of a trade-off between redundancy and reliability. For example, if $g_{2}$ abandons its transmission after detecting the transmission of the duplicate frame by $g_{1}$, none of the 2-hop nodes in the area of $G_{2}-\left(G_{1}\cap G_{2}\right)$ can receive the frame. Otherwise, if both nodes attempt to transmit an identical frame, all of the 2-hop nodes in the area $G_{1}\cap G_{2}$ will receive the duplicate frame. Consequently, the suppression of redundant transmission leads to the degradation of reliability in the traffic dissemination, conversely the reliability cannot be improved without the cost of redundancy. This problem becomes more complex as the number of nodes involved in the partial-overlap increases, which typically occurs in flooding. As shown in Figure 2c, $g_{1}$, $g_{2}$, and $g_{3}$ are closely located, which then makes it difficult to determine which node should be allowed or blocked to transmit a frame in a distributed manner. For example, if $\left(G_{1}\cup G_{3}\right)⊃G_{2}$, it is desirable to allow $g_{1}$ and $g_{3}$ to transmit the frame but to restrict the transmission of $g_{2}$. However, this approach requires the location estimation of the nodes and a signaling mechanism between them, which is difficult to implement without centralized control and incurs a large overhead.

Other serious issues of flooding are collisions and interference due to excessive channel accessing. As shown in Figure 2d, a frame transmitted by the source node *s* may collide with other frames transmitted concurrently by 1-hop nodes (e.g., $g_{1}$, ⋯, $g_{4}$). Furthermore, the frame of a source node cannot be successfully delivered to the 1-hop nodes due to interference by the 2-hop nodes hidden from the source node, e.g., while the source node *s* is transmitting a frame, the 2-hop node $b_{1}$ that is unaware of the transmission of the source node may start to transmit another frame, then node $g_{3}$ probably cannot decode the frame of the source node correctly. Interference may also occur between the transmissions of a 1-hop and 2-hop node as well as between those of the source node and a 2-hop node. For example, if nodes $g_{4}$ and $b_{1}$ are hidden from each other, the transmission of $g_{4}$ may be subjected to interference from that of $b_{1}$, resulting in the failure of the frame delivery from $g_{4}$ to $b_{2}$.

It is clear that the probability of a collision or interference increases as the number of nodes (or the density of nodes) increases. In the case of unicast in WLANs, such collisions and/or interference can be mitigated by adjusting the size of a contention window like binary exponential backoff (BEB) or by employing a handshaking mechanism like request-to-send and clear-to-send (RTS/CTS) exchange. However, in the case of broadcasting, these mechanisms cannot be applied because an acknowledgment (ACK) frame usually does not exist and the receiver is not unique. Besides, due to the absence of an ACK frame, each node (source or relay) cannot recognize whether or not a frame has been successfully delivered to a particular adjacent node.

Because of these reasons, it is challenging to strictly ensure the reliability of flooding. We state the problem of flooding that should be handled in our study as follows:

- Each node should determine whether or not to relay a frame by only overhearing the transmission of adjacent nodes. Thus, a specific criterion for this decision should be established.
- The reliability of traffic dissemination should be provided at an acceptable level without incurring severe duplicate transmissions or increasing the dissemination time significantly.
- As well as collisions, the interference from hidden nodes should be taken into account, which is another main cause of reliability degradation but has not been adequately considered in most existing studies.

### 2.2. Related Work

Flooding mechanisms have been extensively studied in mobile ad hoc networks, especially for the purpose of routing [10,12]. Depending on the criterion or means of broadcast suppression, they can be largely classified as (i) probability-based flooding (PBF), (ii) counter-based flooding (CBF), and (iii) distance-based flooding (DBF). The basic idea behind these mechanisms is as follows: each node determines the relaying (or rebroadcasting) of a frame with a given probability as long as the number of duplicate frames received is smaller than the counter threshold or when the distance between itself and the adjacent relay node is larger than the distance threshold. The value of the probability or the threshold is usually predetermined or fixed in conventional flooding mechanisms [10,12], and so their performance critically depends on one of these predetermined values which is unadaptable to changes in network conditions like the number of nodes or the size of the network. Moreover, the optimal value of probability or threshold may be different for each node.

To overcome these drawbacks, many variants of conventional flooding mechanisms have been proposed. In [13], dynamically adjusting the probability of rebroadcast depending on the density of the nodes (i.e., the number of adjacent nodes) was suggested; the node in a dense area rebroadcasts frames with a low probability to mitigate the redundancy whereas a high value of probability is used in a sparse area to improve the reachability of frames. According to [14,15,16], the probability of rebroadcasting is calculated by considering the additional coverage due to it as well as the degree of connectivity, which is analogous to the number of adjacent nodes. Subsequently, if a frame can be delivered to more adjacent nodes, it is preferentially transmitted. Similarly, the mechanism proposed in our previous work [11] sets the rebroadcast probability as inversely proportional to the number of adjacent nodes but unlike the conventional PBF, it does not drop the duplicate frame but defers the transmission to improve the reliability of flooding.

The performance of CBF can be improved in a similar way. In [17], the value of the counter threshold was adjusted as a function of the number of adjacent nodes. The study in [18] proposed using multiple differentiated levels of the counter threshold depending on the number of adjacent nodes. Meanwhile, there have been several approaches to enhance the performance of DBF. Instead of using a pre-determined threshold of distance, the study in [19] suggested using three differentiated values of the rebroadcast probability depending on the distance from the sender, i.e. if the node is far from (or close to) the source node, it has a higher (or lower) rebroadcast probability. A similar idea was applied to vehicular ad hoc networks [20]; depending on the distance between two adjacent nodes, the rebroadcast probability or delay is controlled so that the traffic dissemination time can be decreased while decreasing the redundancy. It was proposed in [21] to set the distance threshold by considering the probability of transmission failure due to channel error, i.e., a smaller threshold is used if the quality of a wireless channel worsens.

Furthermore, a hybrid approach of combining PBF and CBF has been proposed in the literature. In [22], the rebroadcast probability was set based on the number of duplicate frames received; if the number of duplications was greater or smaller than a predetermined counter threshold, the transmission probability was decreased or increased accordingly. The study in [23] proposed that the node rebroadcasts a frame at a fixed probability if the number of duplications is less than the counter threshold; otherwise, the node drops the frame. It was also proposed in [24,25] to dynamically control the rebroadcast probability depending on the number of duplications such that the probability decreases as the number of duplications increases.

Meanwhile, the broadcast storm problem can be dealt with in a non-probabilistic way. The approach proposed in [26,27,28] uses the network topology information (e.g., list of 1-hop or 2-hop neighbor nodes or their locations) to determine an optimal relay node (called *forwarder*). This approach can quickly extend the coverage of flooding while minimizing the redundant transmissions. However, it leads to an inevitable signaling overhead to acquire the topology information, so it is not suitable when the topology changes frequently or the number of node is large. Furthermore, this approach implicitly assumes that the forwarder successfully deliver a frame to all of its adjacent nodes, which is not always valid due to interference by hidden nodes.

Most of the existing studies attempted to avoid the broadcast storm problem by suppressing the redundant transmissions. However, the excessive suppression may degrade the reachability or reliability of broadcasting, which was referred to as *early die-out* or *premature death* [19,29]. To avoid this problem, it was proposed in [30] that the node relays the frame deterministically (i.e., with the probability of one) as long as the number of adjacent nodes or duplicate frames received is less than a predefined threshold. Another approach was to additionally broadcast the frame after detecting the early die-out. According to [29], if a node does not receive the duplicate frame at least *m* times from its adjacent nodes within a reasonable timeout period *T*, it broadcasts the frame again. The similar idea was proposed in [19]; each node checks whether its adjacent nodes have received the frame by means of *neighbor confirmation* scheme. If some of them did not receive the frame until a certain amount of time *T*, the node broadcasts the frame. The critical issues in [19,29] are that it is hard to determine the optimal values of *m* and *T* in a distributed way and the neighbor confirmation scheme inevitably increases the signaling overhead. The reliable broadcast propagation (RBP) mechanism proposed in [31] employs the implicit ACK and unicast transmission to improve the reliability of broadcast. In [31], each node considers the overheard transmission from 1-hop adjacent node as an implicit ACK and estimates the fraction of acknowledged adjacent nodes. If this fraction is below a given threshold value, the node attempts to retransmit the frame to a specific unacknowledged node as a unicast transmission.

Apart from the flooding mechanisms, many media access control (MAC) protocols for reliable broadcast or multicast in IEEE 802.11 WLANs have been proposed in the literature [32,33,34,35]. They basically exploited the IEEE 802.11 control frames (e.g., ready-to-send (RTS), clear-to-send (CTS), and ACK frames) to improve the reliability. The main issues of these protocols are severe feedback overhead, collision, and the hidden node problem. To reduce the feedback overhead, the negative ACK mechanism was proposed in [32,34], or only a representative receiver (known as *leader*) or multiple ACK-leaders are allowed to transmit the ACK frame [34,35]. Moreover, the transmission sequence or probability of control frames was controlled to mitigate the collision [33,34].

Compared to these existing studies, our one is different due to the following points comprising its advantages:

- Our flooding mechanism provides a unified solution to the broadcast storm problem and early die-out by combining the duplication suppression scheme and re-queuing scheme. The former discards unnecessary duplicate frames in a proactive way, whereas the latter retransmits selected frames in a reactive way. In both schemes, the novel criterion called the duplication ratio is used.
- Unlike the previous flooding mechanisms, the proposed mechanism does not need to tune several control parameters or to determine their optimal values under different network conditions. Therefore, it provides the robust performance to the change of network conditions.
- Our mechanism is completely free from any feedback information or signaling between nodes, so it neither requires a dedicated control frame nor incurs a signaling overhead, which is a crucial feature in the broadcasting to many nodes.

## 3. Duplication Suppression and Re-Queuing

The proposed mechanism consists of two components. The first one is a method of determining the probability of discarding a frame to suppress redundancy and another one is a method attempting the re-queuing of frames to compensate for excessive suppression and unsuccessful transmissions. We first introduce the duplication ratio, which plays a key role in both methods, and then propose two methods.

### 3.1. The Definition and Characteristics of the Duplication Ratio

#### 3.1.1. The Duplication Index and the Duplication Ratio

We first introduce the *duplication index* which indicates whether or not a frame has already been successfully delivered to the adjacent nodes and will be used in estimating the duplication ratio. Let us denote $F_{i,j}$ as a frame with sequence number *j* that node *i* attempts to relay. We also denote $N_{i}$ as the total number of adjacent nodes around node *i* and $n_{i,j}$ as the number of node *i*’s adjacent nodes that have already received the frame $F_{i,j}$ (For the time being, we make the ideal assumption that node *i* can estimate $n_{i,j}$, which is not feasible without a feedback signal between the nodes. Later, we remove this assumption by approximating the duplication ratio). We define the duplication index $I_{i,j}$ for frame $F_{i,j}$ as

(1) $$I_{i,j}=\left\{\begin{array}{cc} 1,\;\; & \mathrm{if}\;\;n_{i,j}\geq \alpha N_{i},\\ 0,\;\; & \mathrm{if}\;\;n_{i,j}<\alpha N_{i}, \end{array}\right.$$

where α(0<α≤1) is a weighting factor referred to as the *duplication-index-decision factor*. Duplication index $I_{i,j}$ has a binary value of 1 or 0 if the level of redundancy is high (i.e., $n_{i,j}\geq \alpha N_{i}$) or not, respectively. Here, α is used to determine the acceptable level of redundancy; in other words, as the value of α approaches 1, $I_{i,j}$ becomes 1 if the frame has been delivered to more adjacent nodes. If $I_{i,j}$ = 1, it is desirable to avoid relaying the frame $F_{i,j}$; otherwise, the frame needs to be relayed. Therefore, a high value of α is helpful to ensure the reliability more strictly, or the redundancy can be mitigated by lowering the value of α.

Now, we introduce the duplication ratio. We denote $c_{i,j}$ as the duplication counter of frame $F_{i,j}$, i.e., the number of times that node *i* received frame $F_{i,j}$ before trying to relay it, and then we define the duplication ratio of node *i*, $DR_{i}\left(\tau \right)$, as

(2) $$DR_{i}\left(\tau \right)=\frac{\sum_{F_{i,j}\in S_{i,\tau }}I_{i,j}}{∥S_{i,\tau }∥},\;\;\;S_{i,\tau }=\left\{F_{i,j}|c_{i,j}=\tau \right\}.$$

Here, $S_{i,\tau }$ is a set of frames in node *i* whose duplication counter is τ and $∥S_{i,\tau }∥$ is the number of elements in set $S_{i,\tau }$. According to (2), $DR_{i}\left(\tau \right)$ is a function of duplication counter τ and is the ratio of the number of frames whose duplication index is 1 to the total number of frames for the given duplication counter τ.

**Figure 3:**
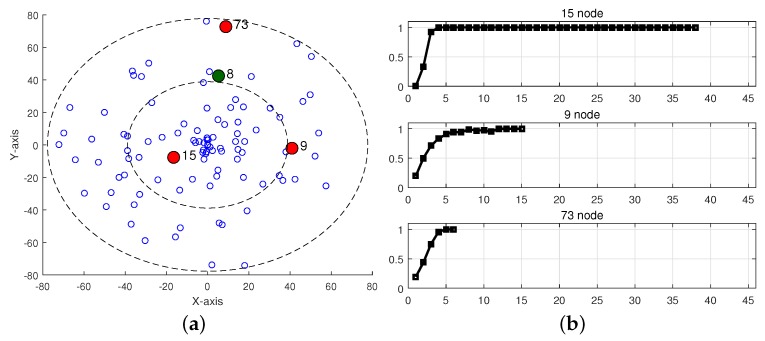
Examples of duplication ratio: (**a**) the network topology to measure the duplication ratio in the simulation, and (**b**) examples of duplication ratio $DR_{i}\left(\tau \right)$ (α = 1) measured in several nodes.

Figure 3b shows the typical value of the duplication ratio obtained from simulation in which, as shown in Figure 3a, a source node was located at the center and 100 nodes were randomly located around the source node within a circular area whose radius is twice of the transmission range of the source node. The duplication ratio in Figure 3b was measured under the condition that all the nodes successfully receive all the frames transmitted by the source node and they relay all the received frames once. The detailed simulation environments are described in Section 4.1. The duplication ratio was measured in three specific nodes: 9, 15, and 73 (marked as red circles in Figure 3a) when α was set as 1. From the results displayed in Figure 3b, we can observe that (i) when the node was located close to the source node (e.g., node 15), $DR_{i}\left(\tau \right)$ sharply increased from 0 and remained close to 1 as τ increased and the maximum value of τ was much larger than the other cases (nodes 9 and 73); (ii) in the case of node 9 located at the edge of carrier sensing range of the source node, $DR_{i}\left(\tau \right)$ approached 1 but its rate of increase decreased as τ increased; (iii) when the node was far away from the source node (e.g., node 73), $DR_{i}\left(\tau \right)$ increased almost linearly from 0.2 to 1 in the short range of τ between 1 and 5. Although the duplication ratios measured in each node were somewhat different, their common features are that $DR_{i}\left(\tau \right)$ is a monotonic increasing function of τ and it approaches 1 as τ increases.

**Figure 4:**
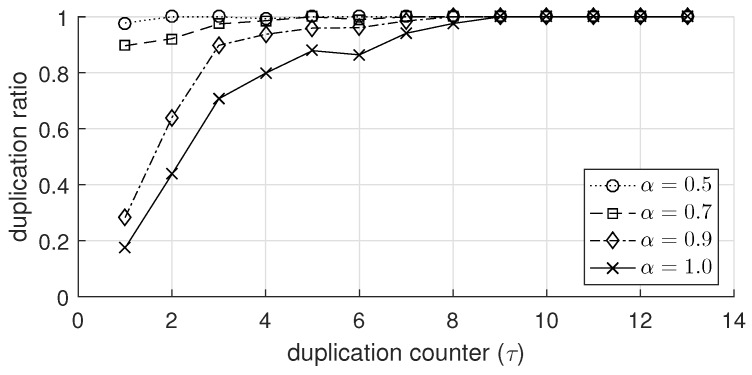
The effect of the duplication-index-decision factor α on the duplication ratio.

On the other hand, Figure 4 shows several values of $DR_{i}\left(\tau \right)$ measured in node 8 (marked as a green circle in Figure 3a) when α had different values. When α was set to 0.5, $DR_{i}\left(\tau \right)$ was almost immune to the change in τ and was very close to 1. As α increased, the value of $DR_{i}\left(\tau \right)$ decreased for a specific τ. The reason is that as α increased, the duplication index in (1) became 1 more conservatively, leading to a decrease in the duplication ratio in (2). Meanwhile, we can define $\tau_{min}$ as the minimum value of τ ensuring that the duplication ratio becomes close to 1, i.e.,

$$\tau_{min}={\mathrm{argmin}}_{\tau }\left(DR_{i}\left(\tau \right)\geq 1-\epsilon \right),\;\mathrm{for}\;\epsilon \left(>\;0\right)\approx 0.$$

As shown in Figure 4, $\tau_{min}$ also increased as α increased. For example, when ϵ = 0.01 and α = 1.0, $\tau_{min}$ was 9, which was somewhat smaller than $N_{i}$ (=14), but $\tau_{min}$ was 2 when α = 0.5. It is worthwhile noting that even in the most conservative case (i.e., α = 1), $DR_{i}\left(\tau \right)$ was very close to 1 for $\tau_{min}\left(<\;N_{i}\right)$, implying that it is possible to disseminate traffic in a sufficiently reliable way even though some adjacent nodes do not relay the frames as long as the duplication counter is greater than $\tau_{min}$.

#### 3.1.2. The Approximation of the Duplication Ratio

The duplication ratio can be obtained based on the duplication index in (1), which relies strongly on assuming that node *i* can estimate $n_{i,j}$. The approach of designing a signaling mechanism to estimate $n_{i,j}$ is not desirable because it tends to incur significant overhead, especially in dense networks, and the feedback information may be stale or incorrect due to the signaling delay. Even with a suitable signaling mechanism, it is difficult to estimate $n_{i,j}$ accurately if any kind of duplication suppression scheme is applied, which may affect $n_{i,j}$ and lead to a distortion in the estimation. To avoid these problems, we propose a simple method of approximating the duplication ratio based on the number of adjacent nodes. It is important to note from Figure 3b that the dominant factor affecting $DR_{i}\left(\tau \right)$ is the maximum value of τ, which mostly depends on $N_{i}$. By taking this point into account, we propose an approximated duplication ratio $ADR_{i}\left(\tau \right)$ defined as

(3) $$ADR_{i}\left(\tau \right)=\delta +\frac{\left(1-\delta \right)\log\left(1+\mu \frac{\tau -1}{N_{i}-1}\right)}{\log(1+\mu )}.$$

This approximation is independent of $n_{i,j}$, but requires the estimation of $N_{i}$, which can be easily estimated by identifying the address fields in the MAC header of a frame received from an adjacent node. In (3), $ADR_{i}\left(\tau \right)$ is derived by modifying the μ-law algorithm [36], which is characterized by two design parameters: δ and μ. Please note that $ADR_{i}\left(1\right)=\delta$, i.e., δ corresponds to the value of the approximated duplication ratio when the node receives a new frame. Therefore, δ can be set to a small value close to 0 from the observations in Figure 3b. Meanwhile, μ determines the slope of $ADR_{i}\left(\tau \right)$.

**Figure 5:**
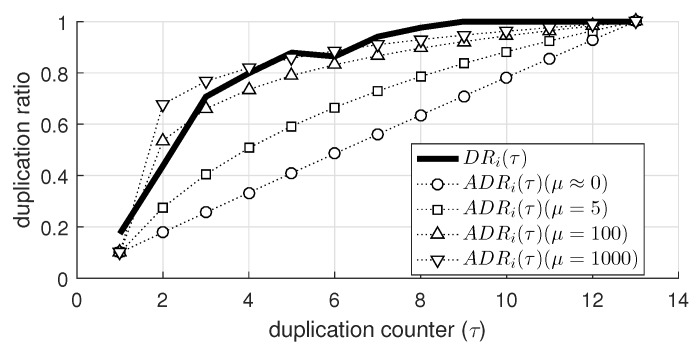
The approximated duplication ratio with several values of μ.

Figure 5 compares the $DR_{i}\left(\tau \right)$ (α = 1) specifically measured at a node 8 (represented as a solid line) to the values of $ADR_{i}\left(\tau \right)$ calculated with different values of μ (represented as dotted lines). Here, δ was set to 0.1. When μ was very small, $ADR_{i}\left(\tau \right)$ linearly increased as τ increased, and $ADR_{i}\left(\tau \right)$ increased and became saturated at 1 more rapidly as μ became larger. We verified through extensive simulations that the performance of the proposed mechanism with $ADR_{i}\left(\tau \right)$ was not much affected by the values of δ and μ as long as δ was close to 0 and μ was sufficiently large.

### 3.2. Redundancy Suppression with Duplication Ratio

Based on the observations on the duplication ratio in Section 3, we designed the redundancy suppression scheme described in Algorithm 1, which is invoked whenever a node receives frame $F_{i,j}$. We consider two kinds of duplicate frames; *arriving duplicate frame (ADF)* and *queued duplicate frame (QDF)*. An arriving frame is defined as ADF if a node has already received one or more frames with the same sequence number as the arriving frame. When a node receives a frame, there may exist a frame in the transmission buffer that has the same sequence number as the arriving frame, which is defined as QDF. The node determines whether the received frame is a duplicate by checking its sequence number. If it is a new frame, the node enqueues it and initializes the duplication counter $c_{i,j}$ to 1 for frames with the sequence number *j*. Otherwise, the node discards the ADF and increases $c_{i,j}$ by 1. Next, the node generates a random variable (rand()) uniformly distributed between 0 and 1 and compares it with the duplication ratio with the duplication counter $c_{i,j}$ ($DR_{i}\left(c_{i,j}\right)$). If $rand\left(\right)\leq DR_{i}\left(c_{i,j}\right)$, the node deletes the QDF from the transmission buffer so that it will not be relayed. According to Algorithm 1, the ADF is definitely discarded whereas the QDF is deleted with the probability of $DR_{i}\left(c_{i,j}\right)$. It is noteworthy that the even a new arriving frame (not ADF) may not be relayed because it can be deleted with a small probability of $DR_{i}\left(1\right)$.

**Algorithm 1:** The redundancy suppression scheme based on the duplication ratio.

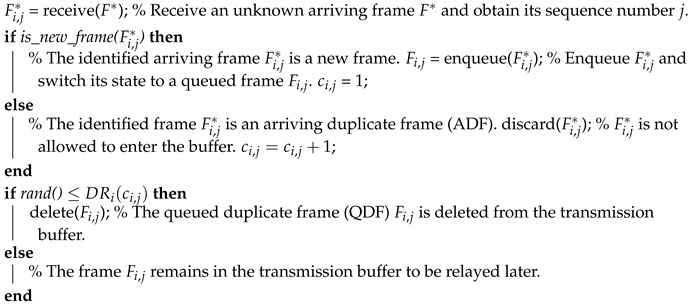



The basic rationale of this operation is that the most of adjacent nodes around to node *i* have already received frame $F_{i,j}$ at the probability of $DR_{i}\left(c_{i,j}\right)$, thus the rebroadcasting of this frame needs to be suppressed at the same probability to reduce the redundancy. We define $P_{tx,i}\left(\tau \right)$ as the probability that frame $F_{i,j}$ could be rebroadcasted without being deleted until its duplication counter reaches τ. Subsequently, $P_{tx,i}\left(\tau \right)$ becomes

(4) $$P_{tx,i}\left(\tau \right)=\prod_{c=1}^{\tau }\left(1-DR_{i}\left(c\right)\right).$$

**Figure 6:**
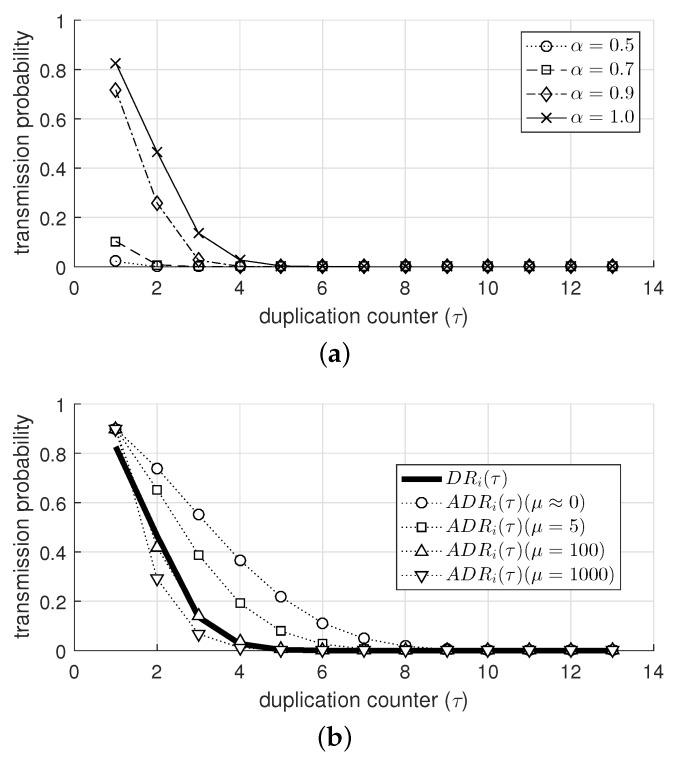
The transmission probability obtained from (**a**) duplication ratio with various values of α and (**b**) approximated duplication ratio with various values of μ.

Figure 6a shows $P_{tx,i}\left(\tau \right)$ with various values of α (as shown in Figure 4). As τ increased, $P_{tx,i}$ exponentially decreased and converged to 0. In addition, the smaller values of α (e.g., 0.7 and 0.5) resulted in a significant decrease in $P_{tx,i}$; in other words, the aggressive suppression of transmission. Similarly, Figure 6b shows $P_{tx,i}$ obtained from $ADR_{i}\left(\tau \right)$ as exhibited in Figure 5. The difference between $P_{tx,i}$ obtained from $DR_{i}\left(\tau \right)$ and $ADR_{i}\left(\tau \right)$ was not notable. Especially when μ was 100, the difference was negligible for the whole range of τ, confirming that the approximated duplication ratio can be practically used in the duplication suppression scheme.

### 3.3. Re-Queuing to Compensate for Delivery Failure

It is clear that all of the frames cannot be successfully delivered to all of the nodes, even with a suitable duplication suppression scheme. We consider three reasons for frame delivery failure: (i) the rebroadcasting of a frame is excessively suppressed, (ii) the error rate of a wireless channel is temporarily high due to fading or shadowing, and (iii) concurrent transmissions by hidden nodes result in a collision and/or interference. To overcome these problems, we propose a compensation scheme called *re-queuing*. This scheme is designed to improve the reliability further by granting an additional chance of transmission to the frame that has been deleted from the transmission buffer due to the duplication suppression scheme or has not been successfully delivered to adjacent nodes. Please note that a frame can be re-queued after it has been transmitted or deleted, thus it can be transmitted up to twice due to the re-queuing scheme. The two main issues in the re-queuing scheme are determining which frames to re-queue and when to do it. Unlike the unicast communication, it is difficult to determine the data frame to be retransmitted because there is no ACK frame in the relayed broadcasting. Moreover, the data frame does not have to be retransmitted immediately after detecting its delivery failure, because it can be recovered by a duplicate transmission from other adjacent nodes. These two issues are discussed in the following subsections.

#### 3.3.1. The Selection Criterion for Re-Queuing Frames

**Figure 7:**
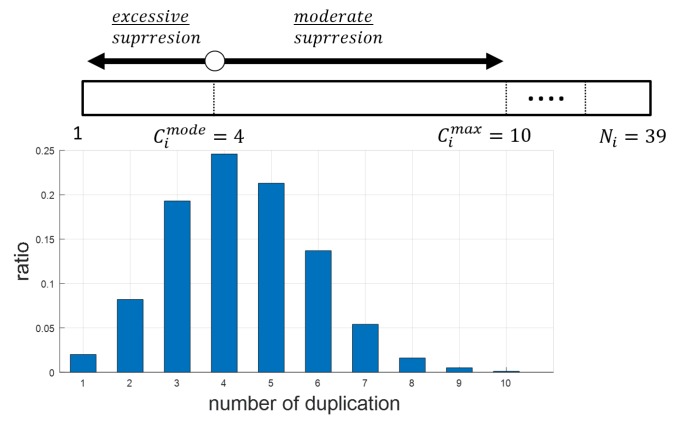
An example of duplication counter distribution.

The re-queuing scheme should be able to determine whether a frame requires a secondary transmission opportunity without resorting to feedback information. For this purpose, we propose using the duplication ratio and the distribution of the duplication counter. Whenever a frame is received, each node collects information about the duplication counter for each of them. Let us denote $C_{i}$ as a set of duplication counters $c_{i,j}$ for all of the frames received by node *i* (Please note that the re-queuing scheme continues to update duplication counter $c_{i,j}$ even after frame $F_{i,j}$ has been transmitted or discarded). Figure 7 shows an example of the distribution of $C_{i}$ in a specific node obtained from simulation when applying our suppression scheme described in Section 3.1. Here, $C_{i}^{mode}$ and $C_{i}^{max}$ are the mode of $C_{i}$ (i.e., the most frequent value of $c_{i,j}$) and the maximum value in $C_{i}$, respectively, and they can be easily estimated with duplication counters. Figure 7 shows that each node usually receives duplicate frames from its adjacent nodes at more or less $C_{i}^{mode}$ times. We can expect that the value of $C_{i}^{mode}$ is comparable to the number of adjacent nodes participating in the relay (referred to as active adjacent nodes) because the proposed duplication suppression scheme allows each node to relay a frame once at most or blocks the relay. By taking these points into account, we introduce $\Delta_{i,j}$ as a criterion to determine whether it is necessary for node *i* to re-queue frame $F_{i,j}$ and define it as the difference between the expected number of active adjacent nodes (${\hat{n}}_{i,j}$) and the mode of $C_{i}$, i.e.,

(5) $$\Delta_{i,j}={\hat{n}}_{i,j}-C_{i}^{mode}.$$

We also propose estimating ${\hat{n}}_{i,j}$ as

(6) $${\hat{n}}_{i,j}=\left\lceil \alpha \times DR_{i}\left(c_{i,j}\right)\times C_{i}^{max}\right\rceil ,$$

where ⌈x⌉ is a round-up function giving the smallest positive integer greater than or equal to *x*. The rationale for estimating ${\hat{n}}_{i,j}$ in (6) is as follows. The duplication ratio can be regarded as the probability that the ratio of the number of adjacent nodes that received frame $F_{i,j}$ to the total number of adjacent nodes ($N_{i}$) is not smaller than α, i.e.,

(7) $$DR_{i}\left(c_{i,j}\right)=Pr\left[\frac{n_{i,j}}{N_{i}}\geq \alpha |c_{i,j}=\tau_{j}\right].$$

Not all of the adjacent nodes attempt to relay frames due to the duplication suppression mechanism, but the maximum number of active adjacent nodes can be considered as $C_{i}^{max}$. Therefore, the product of α, $DR_{i}\left(c_{i,j}\right)$, and $C_{i}^{max}$ can be used as the estimate of ${\hat{n}}_{i,j}$. Please note that the term $DR_{i}$ in (6) can be replaced with an approximated value, i.e., $ADR_{i}$.

By using criterion $\Delta_{i,j}$, node *i* determines whether or not frame $F_{i,j}$ needs to be re-queued. As long as $\Delta_{i,j}\geq 0$, i.e., ${\hat{n}}_{i,j}$ is not smaller than $C_{i}^{mode}$, we can consider that frame $F_{i,j}$ has been relayed several times by its adjacent nodes, so node *i* does not re-queue frame $F_{i,j}$. On the contrary, if $\Delta_{i,j}<$ 0, node *i* starts the re-queuing process for frame $F_{i,j}$. For the purpose of re-queuing, a copy of the QDF needs to be maintained even after it has been discarded or transmitted. It is noteworthy that the re-queuing scheme does not immediately attempt to transmit the frame when detecting $\Delta_{i,j}<$ 0; it waits for a reasonable observation time (denoted as $T_{RQ}$) because node *i* may receive another duplicate frames soon from its adjacent nodes so that $\Delta_{i,j}$ becomes positive. We present a simple example when α = 1, $C_{i}^{mode}=4$, and $C_{i}^{max}=10$, as indicated in Figure 7. Assume that $DR_{i}\left(2\right)$ = 0.25 and $DR_{i}\left(3\right)$ = 0.45 and that node *i* receives frame $F_{i,j}$ twice from its adjacent nodes at a certain time, then ${\hat{n}}_{i,j}$ (= ⌈2.5⌉ = 3) is smaller than $C_{i}^{mode}$ (=4), and so node *i* considers re-queuing frame $F_{i,j}$. However, if node *i* receives a third duplicate frame of $F_{i,j}$ before the observation time expires, then ${\hat{n}}_{i,j}$ increases to 5 (=⌈4.5⌉) and $\Delta_{i,j}>$ 0, so the frame is not re-queued. Lastly, it should be noted that once the frame is determined to be re-queued, it is rearranged in the transmission buffer according to the sequence number so that the re-queued frame can be delivered preferentially.

#### 3.3.2. Frame Observation Period for Re-Queuing

Another issue of re-queuing is how to determine the observation period $T_{RQ}$ properly. If $T_{RQ}$ is set too small, a frame is re-queued unnecessarily; otherwise, if it is set too large, the compensation is delayed for too long. Here, we provide a rough guideline to set $T_{RQ}$ appropriately by following the performance analysis of WLANs presented in [37]. Please note that the objective of this analysis is not to derive an accurate model for evaluating the performance of the re-queuing scheme.

We make the following assumptions: (i) node *i* contends for channel access with $C_{i}^{max}$ adjacent nodes, (ii) all of the nodes can sense the transmissions made by each other and they always have data frames to transmit, and (iii) the size of the contention window is fixed to CW in all of the nodes (Similar to the conventional broadcasting mechanisms, our mechanism works without the ACK frame, and thus the size of the contention window cannot be adapted according to the BEB mechanism). We define $P_{i}^{acc}$ as the probability that at least one node (either node *i* or an adjacent node) attempts to transmit frames, which can be represented from [37] as

(8) $$P_{i}^{acc}=1-{\left(1-\lambda \right)}^{C_{i}^{max}+1},$$

where $\lambda =\frac{2}{CW}$ is the probability that a node accesses the channel to transmit a frame in a given time slot according to the carrier sense multiple access (CSMA). We also define ${\bar{T}}_{tx}$ as the average transmission time of a data frame, regardless of whether the transmission has been successful or not. According to [37], ${\bar{T}}_{tx}$ can be represented as

(9) $$\begin{array}{cc} {\bar{T}}_{tx} & =\frac{1}{P_{i}^{acc}}\left(\left(1-P_{i}^{acc}\right)T_{slot}+P_{i}^{acc}T_{tx}\right),\\ & =\frac{1-P_{i}^{acc}}{P_{i}^{acc}}T_{slot}+T_{tx}, \end{array}$$

where, $T_{slot}$ is the slot time and $T_{tx}$ is the transmission time of data frame including the distributed coordination function inter-frame space (DIFS) time. In (9), the first and second terms represent the idle time due to backoff and the busy time due to data frame transmission, respectively. We set $T_{RQ}$ as the multiplication of ${\bar{T}}_{tx}$ and $C_{i}^{max}$, i.e.,

(10) $$T_{RQ}=C_{i}^{max}{\bar{T}}_{tx}.$$

Considering that node *i* usually receives the duplicate frame $C_{i}^{mode}$ times successfully, rather than $C_{i}^{max}$ times, it seems that $T_{RQ}$ in (10) is somewhat overestimated. However, this is not the case. The frame transmission may fail due to interference by hidden nodes as well as collisions. By taking the unsuccessful transmissions into account, we set $T_{RQ}$ as the expected time of $C_{i}^{max}$ transmissions instead of $C_{i}^{mode}$ transmissions. From (8)–(10), we can see that $T_{RQ}$ mostly depends on $C_{i}^{max}$, which can be easily estimated. For example, it ranges between 4.4 and 13.2 ms for a typical value of $C_{i}^{max}$ between 10 and 30.

### 3.4. The Implementation of the Proposed Mechanism

**Figure 8:**
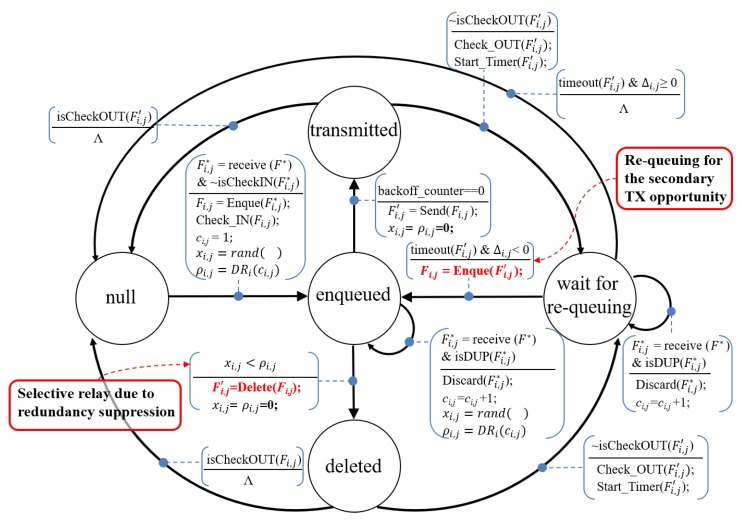
A state diagram of the proposed mechanism consisting of the duplication suppression and re-queuing schemes.

We can implement the overall proposed mechanism as is illustrated in the state diagram in Figure 8. A frame can have the five logical states: null, enqueued, transmitted, deleted, and wait for re-queuing; several operations can be performed depending on the frame state as follows:

1. The null state: This is either the initial or final state of the frame: the former is the state when node *i* has not yet received frame $F_{i,j}$ whereas the latter is when it has finished all of the operations for this frame. If an unidentified frame $F^{*}$ arrives at the node, function receive($F^{*}$) is invoked to identify frame $F_{i,j}^{*}$ by checking its sequence number. Function isCheckIN($F_{i,j}^{*}$) returns true if the frame has already been enqueued more than once. If frame $F_{i,j}^{*}$ is a new frame (i.e., isCheckIN($F_{i,j}^{*}$) is false), then the frame is enqueued and its duplication counter is initialized to 1. Moreover, function check_IN($F_{i,j}$) indicates that the frame whose sequence number is *j* has entered the transmission buffer. After performing these operations, the state of the frame is switched to the enqueued state. Two initialized variables $x_{i,j}$ and $\rho_{i,j}$ denote the random variable and the duplication ratio, respectively, and they are used to determine whether the frame $F_{i,j}$ in the transmission buffer is deleted or not after entering to the enqueued state.
2. The enqueued state: In this state, the frame waits in the buffer for transmission and its state is changed to either the transmitted or deleted state. If the backoff counter becomes 0 and frame $F_{i,j}$ remains in the buffer, it is transmitted by the Send($F_{i,j}$) function. If the node receives another frame $F_{i,j}^{*}$ that is a duplicate (i.e., isDUP($F_{i,j}^{*}$) is true), it discards $F_{i,j}^{*}$ and updates the corresponding $c_{i,j}$, $x_{i,j}$, and $\rho_{i,j}$. Moreover, the node deletes frame $F_{i,j}$ waiting for transmission in the buffer depending on $x_{i,j}$ and $\rho_{i,j}$ (i.e., duplication ratio $DR_{i}\left(c_{i,j}\right)$) using the Delete($F_{i,j}$) function. Please note that the re-queued frame may also be deleted or transmitted again depending on the duplication ratio. Meanwhile, when function Send($F_{i,j}$) or Delete($F_{i,j}$) is called, the state of frame with the sequence number *j* is changed from $F_{i,j}$ to $F_{i,j}^{{}^{\prime }}$, and $x_{i,j}$ and $\rho_{i,j}$ are initialized to 0.
3. The transmitted and deleted states: If the state transition from the transmitted or deleted to the wait for re-queuing state has never happened (i.e., isCheckOUT($F_{i,j}^{{}^{\prime }}$) is false), the re-queuing process is initiated by function Start_Timer($F_{i,j}^{{}^{\prime }}$) and function check_OUT($F_{i,j}^{{}^{\prime }}$) indicates that the frame whose sequence number is *j* has been transmitted or deleted at least once. If isCheckOUT($F_{i,j}^{{}^{\prime }}$) is true, no further operation is performed on the frame and the state is changed to null.
4. The wait for re-queuing state: In this state, the re-queuing procedure controls the frame $F_{i,j}^{{}^{\prime }}$ (a copy of $F_{i,j}$). After the frame observation period has expired (i.e., timeout($F_{i,j}^{{}^{\prime }}$) is true), the state is switched to either enqueued or null depending on the value of $\Delta_{i,j}$. If $\Delta_{i,j}<$ 0, the frame $F_{i,j}^{{}^{\prime }}$ is re-queued to obtain a secondary transmission opportunity; otherwise, the node completes all of the operations for this frame. On receiving the ADF, the node discards it and updates $c_{i,j}$.

## 4. Simulation Study

### 4.1. Simulation Configuration

We implemented the simulator using MATLAB focusing on the physical (PHY) and media access control (MAC) layers of the IEEE 802.11 standard. In the PHY layer, TGn channel model C [38,39] was used as a path loss model. The frequency and bandwidth of the channel were set to 5.25 GHz and 20 MHz, respectively. The carrier sensing threshold, transmission power, and background noise were set to −82, 10, and −100 dBm, respectively. When the minimum receiver sensitivity was −82 dBm, the transmission distance $D_{tx}$ was calculated as 38.9 m, which corresponds to the radius of a 1-hop transmission. We considered transmission failures due to co-channel interference by modeling the bit error rate (BER) according to [40], which considers the punctured convolutional code and several modulation schemes defined in IEEE 802.11n. In each slot time (i.e., 9 μs), our simulator measured the value of the signal-to-interference-and-noise ratio (SINR) from the path loss model and calculated the value of BER. The probability of frame error at each slot time was determined based on the BER and the number of bits per slot time. Please note that the common event-driven network simulator like ns-2 [41] or ns-3 [42] usually calculates the probability of frame error on a per-frame basis by using a simple error model. Therefore, it difficult to accurately model the various effects of interference and frame error, which are essential in this study. Consistent with the IEEE 802.11n standard where the number of spatial streams is one and the guard interval is 800 ns, the transmission rate of all of the nodes was set as 19.5 Mb/s by considering the modulation of QPSK and a coding rate of 3/4. Moreover, we implemented the CSMA features in the MAC layer, the contention window size was set to 15 slots and the frame size to 1 Kbyte.

We considered a basic simulation scenario as follows: a single source node located at the center of the network generates and broadcasts 1 Mbyte of traffic consisting of 1000 frames and all of the other nodes relay the received frames.

To investigate the effect of network scale and node density, we placed nodes (except for the source node) randomly within the network radius of $2D_{tx}$ or $3D_{tx}$, and set the number of nodes $N_{node}$ in the range of 60–140 or 160–240, respectively. In this simulation configuration, the average number of adjacent nodes was between approximately 20 and 50 to ensure that the problems of redundancy, collision, and interference would be severe without a proper duplication suppression scheme.

We considered the following flooding mechanisms for the performance comparison:

- BASE: This is a simple baseline flooding mechanism; all the received duplicate frames are discarded but all the new frames received successfully are transmitted without being suppressed.
- CBF: This is a conventional counter-based flooding scheme and the counter threshold was set to 2 (The threshold value has a significant impact on the performance of CBF. According to [10], its optimal value becomes a lower value when the topology is denser. We also verified that its performance was the best in most simulations when the threshold was 2).
- PBF: As proposed in our previous work [11], this mechanism sets the transmission probability as inversely proportional to the number of adjacent nodes (i.e., $1/N_{i}$). If a duplicate transmission is suppressed, this mechanism defers the transmission of the frame instead of dropping it to improve the reliability of flooding.
- DBF: In this mechanism, each node rebroadcasts the received frames with three differentiated probabilities depending on the distance from the sender. It used three equal distances divided from the maximum transmission distance ($D_{tx}$), as described in [19].
- ${\mathrm{DRBF}}_{\mathrm{RQ}}$, ${\mathrm{ADRBF}}_{\mathrm{RQ}}$: They are the proposed mechanisms implementing the duplication suppression scheme and the re-queuing scheme as proposed in Section 3.2 and Section 3.3, respectively. The duplication ratio in (2) was used in ${\mathrm{DRBF}}_{\mathrm{RQ}}\;\left(\alpha =1.0\right)$ whereas the approximated one in (3) was used in ${\mathrm{ADRBF}}_{\mathrm{RQ}}\;\left(\delta =0.1,\mu =1000\right)$ (We determined the values of δ and μ heuristically after performing many simulations under various configurations). Meanwhile, DRBF and ADRBF denote mechanisms that the re-queuing scheme without the re-queuing scheme.

We observed and compared the performance in various aspects with the indices summarized in Table 1. We repeated the simulations 10 times with a different distribution of nodes and obtained the average values of these performance indices.

### 4.2. The Effect of the Duplication-Index-Decision Factor

**Figure 9:**
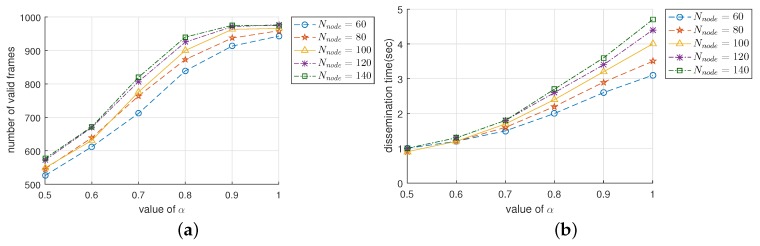
The effect of α on the performance of DRBF in terms of (**a**) the number of valid frames ${\bar{F}}_{val}$ and (**b**) dissemination time ${\bar{T}}_{dis}$.

In this section, we observe the effect of α (duplication-index-decision factor) on the performance of DRBF. Recall that as shown in Figure 4 and Figure 6a, as α decreased, the duplication ratio increased and the transmission of the duplicate frame was aggressively suppressed. Figure 9 shows four performance indices; ${\bar{F}}_{val}$ and ${\bar{T}}_{dis}$, for various values of α ranging from 0.5 to 1 and $N_{node}$ ranging from 60 to 140. First, we observed ${\bar{F}}_{val}$ shown in Figure 9a. When α increased from 0.5 to 0.8, ${\bar{F}}_{val}$ increased almost linearly from approximately 550 to 965. However, the rate of increase in ${\bar{F}}_{val}$ lessened when α exceeded 0.8. In particular, when α≥0.9 and $N_{node}\geq 100$, ${\bar{F}}_{val}$ did not change much and remained at around 965.  Next, we observed from Figure 9b how ${\bar{T}}_{dis}$ changed depending on α and $N_{node}$. As α increased, ${\bar{T}}_{dis}$ increased and its slope also mostly increased. Moreover, the difference between the ${\bar{T}}_{dis}$s with different values of $N_{node}$ was negligible when α was 0.5; but it was remarkably magnified as α increased. For example, when $N_{node}$ was between 60 and 140, ${\bar{T}}_{dis}$ was approximately between 0.98 s and 0.99 s in the case of α = 0.5, but it expanded to between 3.0 s and 4.5 s in the case of α = 1. We can summarize the results in Figure 9 as follows:

- When the value of α is small (e.g., 0.5), the increase in α is considerably effective at improving the reliability in terms of ${\bar{F}}_{val}$ while somewhat degrading the performance of fast traffic dissemination in terms ${\bar{T}}_{dis}$.
- When the value of α is large (e.g., more than 0.9), the increase in α slightly increases ${\bar{F}}_{val}$ but notably increases ${\bar{T}}_{dis}$.
- It is desirable to set α close to 1 for applications that are primarily intended to ensure the reliability strictly (Apart from increasing α, the reliability of flooding can be further improved by adopting a proper coding technique in the application layer (e.g., erasure coding or digital fountain [43])). whereas it is better to set α as a relatively small value if the quality-of-service (QoS) of applications largely depends on the latency.

### 4.3. A Performance Comparison of the Various Flooding Mechanisms

We compare the performance of the proposed mechanisms with those of the existing ones in various aspects.

#### 4.3.1. The Number of Valid Frames and Dissemination Time

**Figure 10:**
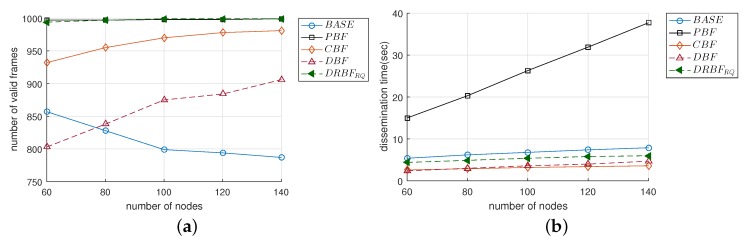
The performance comparison of the flooding mechanisms in terms of (**a**) the number of valid frames (${\bar{F}}_{val}$) and (**b**) the dissemination time (${\bar{T}}_{dis}$).

Figure 10a compares ${\bar{F}}_{val}$ when $N_{node}$ changed from 60 to 140. In the case of BASE, ${\bar{F}}_{val}$ decreased from approximately 860 to 790 as $N_{node}$ increased because of the broadcasting storm problem. On the other hand, the ${\bar{F}}_{val}$ of DBF gradually increased as $N_{node}$ increased. As a result, the ${\bar{F}}_{val}$ of DBF was higher than that of BASE when $N_{node}>$ 80. However, compared to the other mechanisms, the performance of DBF was not acceptable in terms of ${\bar{F}}_{val}$. Except for BASE and DBF, the ${\bar{F}}_{val}$s of the other mechanisms were not much affected by the increase in $N_{node}$ and these values much larger compared to BASE; the ${\bar{F}}_{val}$s were maintained at approximately 998 in the cases of PBF, ${\mathrm{DRBF}}_{\mathrm{RQ}}$, and the ${\bar{F}}_{val}$ of CBF slightly increased from 932 to 981 when $N_{node}$ was increased from 60 to 140. The notable results observed in Figure 10a were as follows: (i) due to the duplication suppression, the reliability of flooding was nearly immune to the change of node density; (ii) the ${\bar{F}}_{val}$ of ${\mathrm{DRBF}}_{\mathrm{RQ}}$ kept strictly close to the ideal; and (iii) ${\mathrm{DRBF}}_{\mathrm{RQ}}$ outperformed CBF that used the optimal value of the counter threshold.

We can see in Figure 10b that the performance of PBF was significantly degraded in terms of ${\bar{T}}_{dis}$, although it seemed to be ideal in terms of ${\bar{F}}_{val}$. Compared to BASE, the ${\bar{T}}_{dis}$ of PBF was greater by 2.8–4.8 times because the latter is designed to achieve high reliability by deferring the transmission of queued duplicate frames instead of deleting them. ${\bar{T}}_{dis}$ increased almost linearly with $N_{node}$. In all of the mechanisms, CBF mostly maintained the smallest value of ${\bar{T}}_{dis}$, which was comparable to the ${\bar{T}}_{dis}$ of DBF. Although the ${\bar{T}}_{dis}$ of ${\mathrm{DRBF}}_{\mathrm{RQ}}$ was not the shortest due to the re-queuing scheme, it was smaller than BASE and PBF by up to 24% and 6.3 times, respectively.

#### 4.3.2. The Number of Duplicate Frames

**Figure 11:**
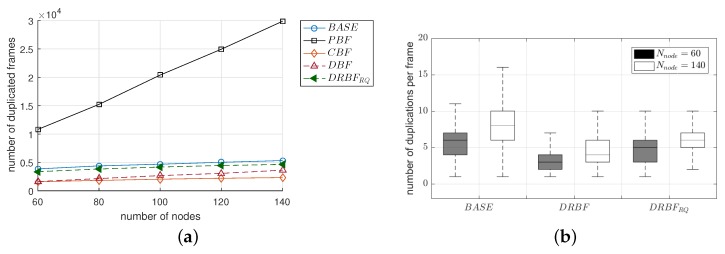
The performance comparison of the flooding mechanisms in terms of (**a**) the number of duplicate frames (${\bar{F}}_{dup}$) and (**b**) the distribution of number of the duplications in all of the nodes.

We investigated the performance in terms of redundancy. As shown in Figure 11a, ${\bar{F}}_{dup}$s in all of the mechanisms linearly increased as $N_{node}$ increased. This result was very similar to ${\bar{T}}_{dis}$ and implies that the transmission of duplicate frames is the main reason for the increase in ${\bar{T}}_{dis}$. More specifically, as $N_{node}$ increased from 60 to 140, the ${\bar{F}}_{dup}$s of BASE and PBF increased approximately from 3800 to 5300 and from 10,800 to 29,900, respectively. Considering that the source node generates only 1000 frames, the nodes in BASE and PBF received many more duplicate frames by up to 5 and 30 times, respectively. Please note that in the case of BASE, even the increase in the number of duplicate frames (Figure 11a) not only increased the traffic dissemination time (Figure 10b) but also degraded the reliability (Figure 10a), thus confirming the necessity of duplication suppression in the flooding. Serious redundancy significantly decreased in ${\mathrm{DRBF}}_{\mathrm{RQ}}$. Compared to PBF, ${\mathrm{DRBF}}_{\mathrm{RQ}}$ decreased ${\bar{F}}_{dup}$ by up to 6.4 times. The ${\bar{F}}_{dup}$ of CBF was the smallest and comparable to that of DBF, but they attained smaller ${\bar{F}}_{val}$s than ${\mathrm{DRBF}}_{\mathrm{RQ}}$, i.e., CBF and DBF cannot assure the high reliability of flooding, despite their lower level of redundancy, compared to ${\mathrm{DRBF}}_{\mathrm{RQ}}$.

We further observed the redundancy in a different aspect. Figure 11b shows the distribution of the number of duplicate frames received in all of the nodes (denoted as $N_{dup}$) in BASE, DRBF, and ${\mathrm{DRBF}}_{\mathrm{RQ}}$. In Figure 11b, the lower and upper bars indicate the 1st and 99th percentiles, respectively, and the bottom, top, and middle line of the box represent the 25th and 75th percentiles, and the median value, respectively. In the case of BASE, $N_{dup}$ was was distributed over a higher and wider range than DRBF and ${\mathrm{DRBF}}_{\mathrm{RQ}}$; when $N_{node}$ was 60, the 25th and 75th percentiles were 4 and 7, respectively, and they extended to 6 and 10 when $N_{node}$ was 140. On the other hand, DRBF and ${\mathrm{DRBF}}_{\mathrm{RQ}}$ moved the distribution of $N_{dup}$ down, as well as its median value. For example, the 75th percentile of $N_{dup}$ was 4 and 6 in DRBF when $N_{node}$ was 60 and 140, respectively, which were equal to those of the 25th percentile in BASE. In DRBF and ${\mathrm{DRBF}}_{\mathrm{RQ}}$, the difference between the 25th and 75th percentile was at least 2. Moreover, we found that the distribution of $N_{dup}$ in ${\mathrm{DRBF}}_{\mathrm{RQ}}$ when $N_{node}$ = 60 was mostly identical to that in DRBF when $N_{node}$ = 140. This implies that the re-queuing scheme in ${\mathrm{DRBF}}_{\mathrm{RQ}}$ has an effect similar to virtually increasing the number of active nodes so that the frame delivery failure can be recovered by the more of adjacent nodes.

#### 4.3.3. Channel Access Contention

**Figure 12:**
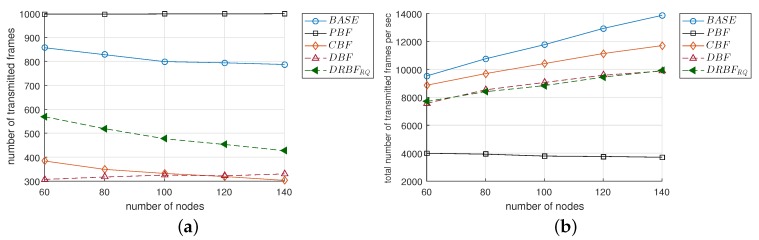
The performance comparison of the flooding mechanisms in terms of (**a**) the number of transmitted frames ${\bar{F}}_{tx}$ and (**b**) the total number of transmitted frames per second ${\bar{R}}_{tx}$.

We observed the performance of the flooding mechanisms from the viewpoint of channel access contention. We first focused on ${\bar{F}}_{tx}$ defined as the average number of frames transmitted per node. Since the channel access contention can be mitigated by restricting each node from sending many duplicate frames, it can be evaluated with ${\bar{F}}_{tx}$. Figure 12a shows that the ${\bar{F}}_{tx}$ of BASE was mostly larger than 800 in the entire range of $N_{node}$. However, in the case of PBF, it was very close to 1000, which means that every node relayed the same number of frames as the total number of frames generated at the source node. Recall that the node in BASE and PBF tries to relay all of the received frames without discarding some of them, thus ${\bar{F}}_{tx}$ becomes equal to ${\bar{F}}_{val}$ for these mechanisms. On the other hand, the other flooding mechanisms decreased ${\bar{F}}_{tx}$ by selectively discarding duplicate frames in the transmission buffer. As $N_{node}$ increased, ${\bar{F}}_{tx}$ gradually decreased in CBF, DRBF, and ${\mathrm{DRBF}}_{\mathrm{RQ}}$, but it rather slightly increased in DBF, which means that DBF less aggressively suppresses duplicate transmissions as the density of nodes increases. By considering both ${\bar{F}}_{tx}$ and ${\bar{F}}_{val}$, we can say that ${\mathrm{DRBF}}_{\mathrm{RQ}}$ is the most effective mechanism because it decreases the channel access contention while providing high reliability of flooding; i.e., compared to BASE and PBF, ${\mathrm{DRBF}}_{\mathrm{RQ}}$ decreased ${\bar{F}}_{tx}$ by 40% and 51% on average, respectively.

Apart from ${\bar{F}}_{tx}$, we evaluated the channel access contention with another performance index. As already confirmed in Figure 10b, the traffic dissemination time was very different especially in PBF, but it was not taken into account in measuring ${\bar{F}}_{tx}$. Therefore, we introduced ${\bar{R}}_{tx}$, which was calculated as the total number of frames transmitted by all of the nodes divided by the traffic dissemination time. Figure 12b shows that unlike the ${\bar{F}}_{tx}$, PBF had the smallest value of ${\bar{R}}_{tx}$, i.e., PBF mitigated the degree of channel access contention by spreading the frame transmissions over a long time. In terms of ${\bar{R}}_{tx}$, the contention of channel access was the most severe in BASE. The ${\bar{R}}_{tx}$ of DBF was comparable to ${\mathrm{DRBF}}_{\mathrm{RQ}}$. Compared to BASE and CBF, ${\mathrm{DRBF}}_{\mathrm{RQ}}$ decreased ${\bar{R}}_{tx}$ by 24% and 14% on average, respectively, thus confirming that the proposed schemes are effective not only at reducing redundancy but also at mitigating the channel access contention.

### 4.4. The Effect of Re-Queuing Scheme

We evaluated the effect of re-queuing scheme by comparing the performance of DRBF (ADRBF) and ${\mathrm{DRBF}}_{\mathrm{RQ}}$ (${\mathrm{ADRBF}}_{\mathrm{RQ}}$).

**Figure 13:**
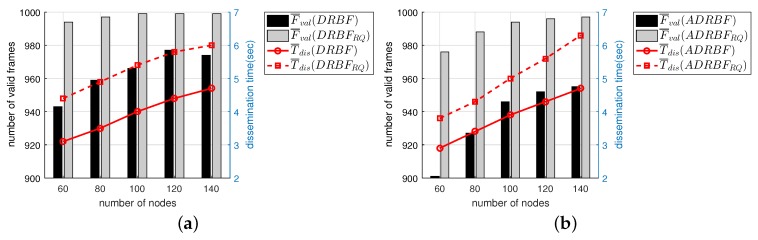
Perfomance comparions of (**a**) DRBF and ${\mathrm{DRBF}}_{\mathrm{RQ}}$ (**b**) ADRBF and ${\mathrm{ADRBF}}_{\mathrm{RQ}}$.

Figure 13a compares ${\bar{F}}_{val}$ and ${\bar{T}}_{dis}$ of DRBF and ${\mathrm{DRBF}}_{\mathrm{RQ}}$. In the case of DRBF, ${\bar{F}}_{val}$ increased from 943 to 977 until $N_{node}$ increased from 60 to 120 but slightly decreased if $N_{node}$ exceeded 120. However, when the re-queuing scheme was applied, ${\bar{F}}_{val}$ of ${\mathrm{DRBF}}_{\mathrm{RQ}}$ was hardly affected by $N_{node}$, it was maintained between 994 and 999. These results mean that the re-queuing scheme is more effective when the density of node is low. By comparing the results of ${\bar{F}}_{val}$ and ${\bar{T}}_{dis}$, we found the trade-off between transmission reliability and traffic dissemination time, The values of ${\bar{T}}_{dis}$ in DRBF and ${\mathrm{DRBF}}_{\mathrm{RQ}}$ increased almost linearly as $N_{node}$ increased, but ${\bar{T}}_{dis}$ of ${\mathrm{DRBF}}_{\mathrm{RQ}}$ was larger than that of DRBF by 28%–42% for the entire range of $N_{node}$ between 60 and 140. Similarly, we can observe the effect of re-queuing scheme applied to ADRBF from Figure 13b. Due to the approximation error of duplication ratio, ${\bar{F}}_{val}$ of ADRBF was somewhat smaller than that of DRBF; it ranged between 901 and 955 when $N_{node}$ is between 60 and 140. Compared to ${\mathrm{DRBF}}_{\mathrm{RQ}}$, the gain of re-queuing in terms of ${\bar{F}}_{val}$ was magnified in ${\mathrm{ADRBF}}_{\mathrm{RQ}}$; ${\bar{F}}_{val}$ of ${\mathrm{DRBF}}_{\mathrm{RQ}}$ was greater than that of DRBF by 22–51, but the difference between ${\bar{F}}_{val}$s in ${\mathrm{ADRBF}}_{\mathrm{RQ}}$ and ADRBF was increased up to 75. On the other hand, there was little difference between ${\bar{T}}_{dis}$s in DRBF and ADRBF, implying that the approximation of duplication ratio has a marginal effect on ${\bar{T}}_{dis}$ when the re-queuing scheme was not used. However, if the re-queuing scheme was applied, ${\mathrm{ADRBF}}_{\mathrm{RQ}}$ had lower ${\bar{T}}_{dis}$ than ${\mathrm{DRBF}}_{\mathrm{RQ}}$ by 0.6 s when $N_{node}$ was 60, but ${\bar{T}}_{dis}$ of ${\mathrm{ADRBF}}_{\mathrm{RQ}}$ was greater than that of ${\mathrm{DRBF}}_{\mathrm{RQ}}$ by 0.3 s when $N_{node}$ was 140.

### 4.5. The Effect of the Duplication Ratio Approximation

In this section, we evaluated the effectiveness of the duplication ratio approximation by comparing the performance deviations between DRBF and ADRBF and between ${\mathrm{DRBF}}_{\mathrm{RQ}}$ and ${\mathrm{ADRBF}}_{\mathrm{RQ}}$. For this purpose, we introduced two performance indices: $G({\bar{F}}_{val})$ and $G({\bar{T}}_{dis})$, defined as,

$$G\left({\bar{F}}_{val}\right)=\frac{{\bar{F}}_{val}\;\mathrm{of}\;\;\mathrm{ADRBF}}{{\bar{F}}_{val}\;\mathrm{of}\;\;\mathrm{DRBF}},\;\;G\left({\bar{T}}_{dis}\right)=\frac{{\bar{T}}_{dis}\;\mathrm{of}\;\;\mathrm{ADRBF}}{{\bar{T}}_{dis}\;\mathrm{of}\;\;\mathrm{DRBF}}.$$

**Figure 14:**
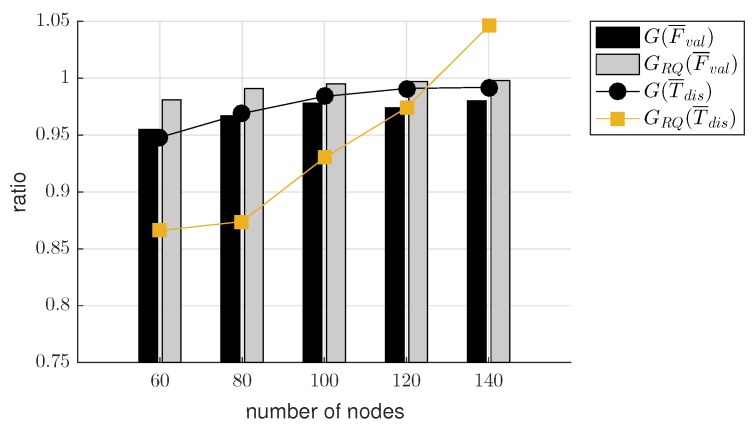
The effect of the duplication ratio approximation on the number of valid frames (${\bar{F}}_{val}$) and traffic dissemination time (${\bar{T}}_{dis}$).

Similar to $G({\bar{F}}_{val})$ and $G({\bar{T}}_{dis})$, we defined $G_{RQ}\left({\bar{F}}_{val}\right)$ and $G_{RQ}\left({\bar{T}}_{dis}\right)$ by considering the performance of ${\mathrm{DRBF}}_{\mathrm{RQ}}$ and ${\mathrm{ADRBF}}_{\mathrm{RQ}}$. Figure 14 shows these four performance indices measured when $N_{node}$ ranged from 60 to 140. As $N_{node}$ was increased, the values of $G({\bar{F}}_{val})$ and $G_{RQ}\left({\bar{F}}_{val}\right)$ (represented as bars in Figure 14) increased from 0.955 and 0.981 to 0.980 and 0.998, respectively; $G_{RQ}\left({\bar{F}}_{val}\right)$ was slightly higher and closer to 1 than $G({\bar{F}}_{val})$. Meanwhile, for the whole range of $N_{node}$, the values of $G({\bar{T}}_{dis})$ and $G_{RQ}\left({\bar{T}}_{dis}\right)$ (represented as lines in Figure 14) were between 0.948 and 0.992 and between 0.866 to 1.046, respectively. From these observations, we can derive the following conclusions:

- The average values of $G({\bar{F}}_{val})$ and $G_{RQ}\left({\bar{F}}_{val}\right)$ were 0.971 and 0.993, respectively, confirming that the reliability of flooding hardly deteriorated due to the approximation error of the duplication ratio. Moreover, its influence diminished as the node density increased or the re-queuing scheme was applied, i.e., $G\left({\bar{F}}_{val}\right)<G_{RQ}\left({\bar{F}}_{val}\right)\approx$ 1.
- The average value of $G({\bar{T}}_{dis})$ was 0.977, meaning that the average traffic dissemination time of ADRBF is comparable to that of DRBF, i.e., the approximation of the duplication ratio has a negligible effect on the traffic dissemination time. Even with the approximation error, ${\bar{T}}_{dis}$ of ${\mathrm{ADRBF}}_{\mathrm{RQ}}$ was usually smaller or slightly higher than ${\mathrm{DRBF}}_{\mathrm{RQ}}$; i.e., the average value of $G_{RQ}\left({\bar{T}}_{dis}\right)$ was 0.938.
- The duplication ratio DR(τ) in (2) can be effectively approximated as ADR(τ) in (3) without severely degrading the performance in terms of ${\bar{F}}_{val}$ and ${\bar{T}}_{dis}$.

### 4.6. The Effect of the Node Mobility

**Figure 15:**
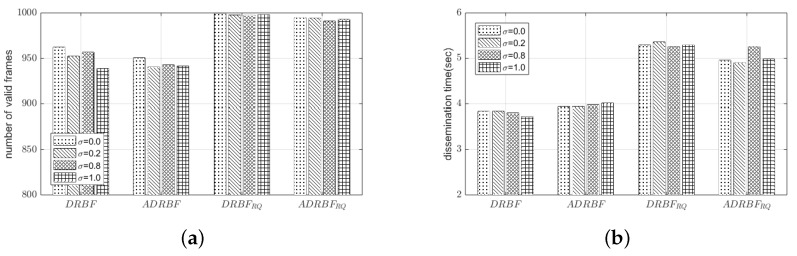
The performance comparison of the proposed flooding mechanisms under the moving scenario: (**a**) the number of valid frames ${\bar{F}}_{val}$ and (**b**) the dissemination time ${\bar{T}}_{dis}$.

We performed the simulation under the scenario where some nodes move. To model the mobility of the node, we introduced the following parameters; the fraction of mobile nodes among the whole nodes (σ), the speed (*v*) and direction (θ) of movement. We considered several values of σ ranging from zero to one to observe the effect of the degree of node mobility. Once a node is determined as a mobile node, its moving speed (*v*) and direction (θ) were randomly determined initially and maintained as constant until the end of simulation time. For each mobile node, we set the value of *v* with a random variable uniformly distributed between 1 and 4 m/s by considering the speed of walking or running, which is reasonable for the service scenario shown in Figure 1. Similarly, we set the value of θ randomly between 0 and 2π. Figure 15 shows ${\bar{F}}_{val}$ and ${\bar{T}}_{dis}$ of our proposed mechanisms when $N_{node}$ was 100 and σ was 0.0, 0.2, 0.8 and 1.0. In all the mechanisms (DRBF, ADRBF, ${\mathrm{DRBF}}_{\mathrm{RQ}}$, and ${\mathrm{ADRBF}}_{\mathrm{RQ}}$), both values of ${\bar{F}}_{val}$ and ${\bar{T}}_{dis}$ were hardly affected by the change of σ, i.e., the performance of the proposed mechanism was hardly degraded due to the node mobility. Meanwhile, the results in Figure 15 confirm again that (i) the re-queuing scheme is effective to improve the reliability of flooding at the cost of traffic dissemination time; compared to DRBF and ADRBF, ${\mathrm{DRBF}}_{\mathrm{RQ}}$ and ${\mathrm{ADRBF}}_{\mathrm{RQ}}$ increased ${\bar{F}}_{val}$ by about 50 frames and also increased ${\bar{T}}_{dis}$ by about 1.5 and 0.6 s, respectively, (ii) ${\mathrm{ADRBF}}_{\mathrm{RQ}}$ compromises the reliability and delay of flooding; compared to ${\mathrm{DRBF}}_{\mathrm{RQ}}$, ${\mathrm{ADRBF}}_{\mathrm{RQ}}$ decreased ${\bar{F}}_{val}$ by only 1.3% but decreased ${\bar{T}}_{dis}$ by more than 18%.

### 4.7. The Effect of the Network Scale

**Figure 16:**
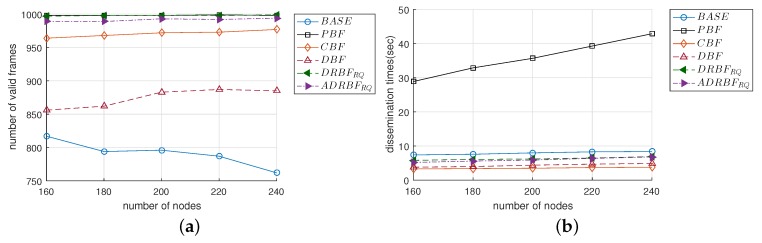
The performance comparison of the flooding mechanisms when the network radius was extended to 3$D_{tx}$: (**a**) the number of valid frames ${\bar{F}}_{val}$ and (**b**) the dissemination time ${\bar{T}}_{dis}$.

The simulation described in this section was performed to investigate the effect of the network scale. The network radius in the previous simulations was $2D_{tx}$, which was extended to $3D_{tx}$ and $N_{node}$ was set between 160 and 240 in this simulation (Considering the possible applications of relayed broadcasting, the radius of a network will typically be two or three times the transmission range of the source node). Figure 16a shows that as $N_{node}$ increased, the ${\bar{F}}_{val}$ of BASE decreased from 817 to 762, but the ${\bar{F}}_{val}$ of DBF increased from 856 and 885, which was greater than that of BASE by 5–16%. Except for these two mechanisms, ${\bar{F}}_{val}$s of the other mechanisms were largely constant even though $N_{node}$ changed. PBF maintained the highest value of ${\bar{F}}_{val}$, which was around 998 regardless of $N_{node}$. The average values of ${\bar{F}}_{val}$ for DRBF and ADRBF were 970 and 947, respectively, but they increased to 998 and 991 when the re-queuing scheme was applied in ${\mathrm{DRBF}}_{\mathrm{RQ}}$ and ${\mathrm{ADRBF}}_{\mathrm{RQ}}$.

As shown in Figure 16b, ${\bar{T}}_{dis}$ for all of the mechanisms linearly increased with $N_{node}$. The ${\bar{F}}_{val}$ of PBF was the highest, which was greater than that of BASE by 3.9–5.1 times. The average values of ${\bar{T}}_{dis}$ for the other mechanisms were not much different: 3.55, 4.38, 4.78, 4.57, 6.32, and 5.99, in CBF, DBF, DRBF, ADRBF, ${\mathrm{DRBF}}_{\mathrm{RQ}}$, and ${\mathrm{ADRBF}}_{\mathrm{RQ}}$, respectively. It was interesting that when compared to DRBF and ${\mathrm{DRBF}}_{\mathrm{RQ}}$, ${\bar{T}}_{dis}$ somewhat decreased in ADRBF and ${\mathrm{ADRBF}}_{\mathrm{RQ}}$. Compared to PBF, the average value of ${\bar{F}}_{val}$ for ${\mathrm{ADRBF}}_{\mathrm{RQ}}$ was 99.3%, but that of ${\bar{T}}_{dis}$ remarkably decreased by 5.5–6.3 times. This result supports the conclusion that the approximated duplication ratio along with the re-queuing scheme is very effective at decreasing the dissemination time without compromising the reliability. By comparing the results in Figure 16 with those in Figure 10 where the network radius was $2D_{tx}$, we can confirm that the outstanding performance of the proposed mechanism is maintained regardless of the network scale.

### 4.8. The Performance Evaluation of the Flooding Mechanism Reliability

In the last simulation, we investigated the reliability of several flooding mechanisms in depth. So far, we have evaluated their reliability with the performance index ${\bar{F}}_{val}$. Since it is an average value when considering all of the nodes, ${\bar{F}}_{val}$ is not effective for observing how many nodes receive how many valid frames. By considering this point, we introduced another performance index $R_{val}\left(p\%\right)$, defined as the ratio of the nodes that received the valid frames more than *p*% of the total nodes. For example, if 80 nodes among 100 nodes received valid frames (that are neither duplicated nor corrupted) more than 95%, $R_{val}\left(95\%\right)$ becomes 0.8. Please note that $R_{val}$ is analogous to the complementary cumulative distribution of the number of valid frames.

**Figure 17:**
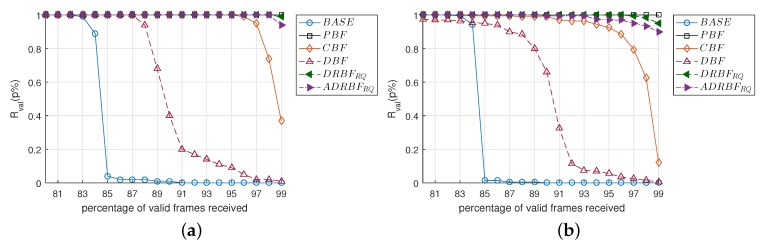
The comparison of flooding reliability in terms of $R_{val}\left(n\%\right)$ when (**a**) the number of nodes is 100 and the network radius is $2D_{tx}$, (**b**) the number of nodes is 200 and the network radius is $3D_{tx}$.

Figure 17a shows $R_{val}\left(p\%\right)$ for various values of *p* ranging from 80 to 99% when $N_{node}$ was 100 and the network radius was $2D_{tx}$. The $R_{val}$ of BASE was smaller than 0.04 when p≥ 85%, whereas that of PBF was 1 even when *p* = 99%, i.e., PBF could ensure the reliability strictly in all of the nodes but nearly no nodes could receive more than 85% of the frames in BASE. The $R_{val}$ of DBF sharply decreased from 0.94 to 0.2 when *p* was increased from 88 to 91%, and it gradually decreased when p> 91%. In the case of CBF, $R_{val}$ suddenly decreased from 0.95 to 0.37 when *p* was increased from 97 to 99%. However, due to the re-queuing scheme, the $R_{val}\left(99\%\right)$ of ${\mathrm{DRBF}}_{\mathrm{RQ}}$ and ${\mathrm{ADRBF}}_{\mathrm{RQ}}$ maintained 0.99 and 0.94, which were greater than that of CBF by 2.7 and 2.5 times, respectively.

We repeated the simulation by increasing $N_{node}$ to 200 and the network radius to $3D_{tx}$ and obtained $R_{val}$, as shown in Figure 17b. When compared to Figure 17a, the performance of BASE and PBF were not much changed. The outstanding performance of $R_{val}$ was still maintained in ${\mathrm{ADRBF}}_{\mathrm{RQ}}$; i.e., even when *p* = 98% and 99%, the $R_{val}\left(p\%\right)$ of ${\mathrm{ADRBF}}_{\mathrm{RQ}}$ were 0.94 and 0.90, whereas those of CBF were only 0.6 and 0.1, respectively. These results in Figure 17 reconfirm that the re-queuing scheme is essential for ensuring the strict reliability and that the approximation error in the duplication ratio does not lead to a considerable degradation in performance if the re-queuing scheme is applied.

## 5. Conclusions

In this paper, we proposed an efficient flooding mechanism aimed at the fast and reliable dissemination of traffic for IoT services. By combining two schemes of duplication suppression and re-queuing and using the duplication ratio, the proposed mechanism selectively discards duplicate frames in the transmission buffer to avoid unnecessary redundant transmissions and at the same time, it compensates for the delivery failure of frames due to excessive suppression, collisions, and/or interference. As a result, it considerably decreased the time of traffic dissemination without compromising its reliability. Moreover, we proposed a practical method of approximating the duplication ratio based on the number of adjacent nodes and verified that the outstanding performance of the proposed mechanism was maintained even with the approximated duplication ratio. For future work, we will extend the proposed mechanism by considering diverse traffic and multiple traffic source nodes in the realistic IoT scenarios and rapid topology change in vehicular ad hoc networks or flying ad hoc networks.

## Figures and Tables

**Table 1:** The performance indices and their descriptions.

Symbol	Description
${\bar{F}}_{val}$	The average number of valid frames per node, i.e., frames received in each node without corruption or duplication.
${\bar{F}}_{tx}$	The average number of frames transmitted per node
${\bar{F}}_{dup}$	The average number of duplicate frames received per node without corruption.
${\bar{T}}_{dis}$	The traffic dissemination time defined as the time from the initial broadcast of the source node until all of the nodes stop relaying.
${\bar{R}}_{tx}$	The total number of frames transmitted per second in the whole network.
G(X)	The performance value of ADRBF divided by that of DRBF in terms of performance index *X*
$G_{RQ}\left(X\right)$	The performance value of ${\mathrm{ADRBF}}_{\mathrm{RQ}}$ divided by that of ${\mathrm{DRBF}}_{\mathrm{RQ}}$ in terms of performance index *X*
$R_{val}\left(p\%\right)$	The fraction of nodes that received valid frames more than *p*%.
